# Endophytic actinomycetes as a reservoir of novel chemotherapeutic leads against multidrug-resistant cancer

**DOI:** 10.3389/frai.2026.1974583

**Published:** 2026-10-01

**Authors:** Dhanvee Ganatra, Mahendrapal Singh Rajput, Gaurav Sanghvi

**Affiliations:** Department of Microbiology, Faculty of Science, Marwadi University, Rajkot, Gujarat, India

**Keywords:** ABC transporters, anticancer activity, endophytic actinomycetes, multidrug resistance, resistance-modulating agents, secondary metabolites, translational drug discovery

## Abstract

Multidrug resistance (MDR) has emerged as a major barrier to effective cancer control, undermining the efficacy of chemotherapy, targeted agents and, increasingly, immunotherapies. This review synthesizes current knowledge on the molecular and cellular bases of MDR in cancer, including ABC transporter–mediated drug efflux, enhanced DNA damage responses, survival-signaling rewiring, tumor-microenvironmental protection and cancer stem cell–driven plasticity. Particular emphasis is placed on endophytic actinomycetes and their metabolites as an underexploited reservoir of MDR modulating chemotypes with potent cytotoxic, resistance-reversal and microenvironment-modulating activities. By integrating recent mechanistic and pharmacological data, the review first delineates the crisis of drug-resistant cancer, then catalogs actinomycete-derived metabolites with documented efficacy against resistant cell lines, including those that inhibit efflux pumps, restore pro-apoptotic signaling or interfere with angiogenesis, epithelial–mesenchymal transition and stemness traits. Finally, translational challenges, such as suboptimal pharmacokinetics, off-target toxicity, herb–drug interactions and the paucity of biomarker-driven clinical trials, are critically examined alongside emerging strategies involving semi-synthetic optimization, nanocarrier delivery and rational combination regimens. A forward-looking discovery pipeline is proposed that couple’s genome mining of endophytic actinomycetes with systematic MDR-mechanism profiling and medicinal chemistry, aiming to prioritize the most promising metabolites for preclinical and clinical development as next-generation MDR-targeted therapeutics.

## 1 Introduction

Cancer remains one of the most formidable global health challenges. Conventional chemotherapeutic and targeted therapies often lead to initial success (Benjamin and Hsu, 2025; Harrold et al., 2024). However, many cancers eventually develop resistance, limiting long-term efficacy and leading to treatment failure (Garg et al., 2024; Ge et al., 2025). This multidrug resistance (MDR) in cancer poses an urgent challenge to improve therapeutic outcomes.

MDR arises through various mechanisms. Cancer cells can over-express drug efflux transporters, alter drug targets, enhance DNA repair, and adapt apoptotic and survival pathways (Halder, 2025). These adaptations allow malignant cells to survive despite high doses of cytotoxic or targeted agents. Many metabolites derived from actinomycetes show significant cytotoxic activity toward different cancer cells, usually in nanomolar to micromolar concentrations.

Activity against the resistant model some compounds can overcome multidrug resistant (MDR) cancer cells, demonstrating the capability to circumvent conventional resistance mechanisms. Proof of mechanism Metabolites exhibit various mechanisms of action, such as induction of apoptosis, inhibition of efflux pumps, DNA damage response, and microenvironmental activities. Because such resistance often involves multiple molecular pathways simultaneously, single-target therapies often fail to restore sensitivity (Garg et al., 2024; Ge et al., 2025; Labrie et al., 2022).

Natural products have emerged as valuable resources in the search for novel MDR modulators. Compared with synthetic small molecules, natural compounds often act on multiple targets or pathways (Pandey and Gupta, 2024). This pleiotropic action may help overcome complex resistance mechanisms in cancer cells. The recent improvement in technologies for isolation and structural elucidation further expands the potential of natural products for MDR-targeted drug discovery (Zou et al., 2024; Bhusare et al., 2024; Chen T. et al., 2024).

Among natural sources, bacteria of the phylum *Actinomycetes*, especially those living as endophytes, represent a vast, underexplored reservoir of natural compounds. Actinomycetes are known for their remarkable capacity to produce structurally diverse secondary metabolites (Selim et al., 2021). Many of these metabolites display bioactivities including antimicrobial, immunomodulatory, enzyme inhibition, and antitumor properties. The discovery pipeline for anticancer drugs still draws heavily on actinomycetes-derived compounds (Khan et al., 2023; Xu et al., 2023).

Endophytic actinomycetes (those that reside inside plant tissues without harming the host) offer additional advantages. Their ecological niche within plants exposes them to unique selective pressures. These pressures often drive the production of novel metabolites that differ structurally and functionally from those produced by soil- or marine-derived actinomycetes (Narayanan and Glick, 2022; Shanthi, 2021). Recent evidence supports that endophytic *streptomycetes* can yield compounds with potent anticancer potential, making them promising candidates for further drug development (Sivalingam et al., 2024).

A systematic review of actinomycetes-derived secondary metabolites between 2019 and 2024 identified multiple structural classes, polyketides, non-ribosomal peptides, alkaloids, and terpenoids, which exhibit potent anticancer effects via diverse mechanisms (Rui et al., 2025). Some metabolites induce apoptosis, inhibit proliferation, and disrupt the tumor microenvironment. Others modulate signaling pathways relevant to oncogenesis and metastasis (Rui et al., 2025; Hassan et al., 2023; Norbakhsh et al., 2022).

Despite progress, the potential of actinomycetes, especially endophytic strains, remains underexplored in the context of reversing MDR in cancer. Previous reviews on natural products for MDR focused mostly on well-known phytochemicals such as curcumin, resveratrol, and baicalein (Chen T. et al., 2024). These reviews rarely emphasize microbial metabolites or deep-dive into actinomycetes.

Therefore, this review aims to (a) present an updated overview of antitumor secondary metabolites from actinomycetes, (b) evaluate their potential for overcoming drug resistance in cancer, and (c) propose strategies for targeted bioprospecting of endophytic actinomycetes as next-generation MDR therapeutics.

## 2 Endophytic Actinomycetes: a reservoir for chemotherapeutic novelty

### 2.1 Ecology and chemical diversity of endophytic actinomycetes

Endophytic actinomycetes occupy a distinctive ecological niche inside healthy plant tissues, where they form largely symptomless associations with roots, stems, leaves and reproductive organs (Kanjanamaneesathian, 2021). Unlike rhizosphere or bulk-soil actinomycetes, these endophytes experience strong selection from plant defenses, limited nutrient availability and competition with other endophytes, which is thought to drive the evolution of specialized biosynthetic gene clusters (BGCs) and unique secondary metabolites (Zhang Z. et al., 2024). Studies of medicinal plants across diverse regions consistently report high isolation frequencies of endophytic actinomycetes, often dominated by Streptomyces but including “rare” genera such as *Actinoallomurus, Micromonospora* and *Actinomadura*, underscoring that plants act as micro-habitats for actinomycete lineages that are difficult to recover from soil alone (Helmi, 2025; Thapa, 2024).

Ecological surveys indicate that the composition and activity of endophytic actinomycete communities depend on host species, organ type and environmental context. For example, work on *Mentha longifolia* and *Lonicera nummulariifolia* identified multiple endophytic actinobacteria that produced metabolites with antibacterial, antifungal and cytotoxic activities, suggesting a role in host defense and adaptation to local stresses (Hatami et al., 2025). Earlier studies on medicinal plants from the Panxi plateau showed that a large proportion of endophytic actinomycetes carried genes encoding type I and type II polyketide synthases and non-ribosomal peptide synthetases, indicating a substantial hidden capacity for secondary-metabolite biosynthesis (Hu et al., 2023). These findings parallel broader biogeographic analyses where actinomycete diversity is shaped by soil pH, organic matter, climate and vegetation, and they support the idea that plant-associated niches, particularly in underexplored or extreme environments, are hotspots for novel actinomycete BGCs (Hatami et al., 2025; Hu et al., 2023).

Chemically, metabolites from endophytic actinomycetes span nearly all major natural-product classes associated with soil actinomycetes, but often with unusual substitution patterns or hybrid scaffolds (Chen T. et al., 2024; Hassan et al., 2023). Systematic reviews of actinomycete-derived antitumor metabolites between 2019 and 2024 show that polyketides, non-ribosomal peptides, alkaloids and terpenoids dominate the structural space, many of which exhibit potent antibacterial, antifungal or antineoplastic activity at nano- to micromolar concentrations. Classic examples include enediyne antibiotics, anthracyclines and chromopeptide lactones, while recent explorations of endophytic strains have yielded novel compounds from rare genera such as *Actinoallomurus* and *Micromonospora* that differ significantly from *Streptomyces*-derived metabolites. The frequent detection of enediyne-associated PKSE genes and other unusual BGCs in endophytic and “rare” actinomycetes supports the view that these taxa are particularly enriched in chemistry that is either absent or extremely rare among soil counterparts (Chen T. et al., 2024; Rui et al., 2025; Hassan et al., 2023; Hatami et al., 2025).

Several large-scale isolation programs highlight the scale of chemical diversity accessible from endophytic actinomycetes. A landmark study that screened over 1,000 root-associated actinomycete isolates reported high hit-rates for bioactive extracts and led to the discovery of multiple structurally novel metabolites, many with antibiotic or cytotoxic properties (Chinnathambi et al., 2023). More recent metabolomics-guided surveys of plant endophytes show that a high proportion of isolates produce multiple bioactivities, such as antimicrobial, antioxidant and enzyme-inhibitory effects, reflecting broad biosynthetic repertoires that can be tuned by culture conditions (De Simeis and Serra, 2021). When integrated with genome mining, these metabolomic profiles reveal extensive cryptic BGCs that are silent under standard fermentation but can be activated by co-culture, epigenetic modifiers or engineering of regulatory networks, offering substantial scope for expanding the known chemical space of actinomycete metabolites (Khalid et al., 2024).

From a drug-discovery perspective, the ecological and chemical features of endophytic actinomycetes are highly advantageous for anticancer and anti-MDR research (Sivalingam et al., 2024). Co-evolution with plant hosts likely selects for metabolites that modulate signaling, oxidative stress and defense pathways, which overlap mechanistically with apoptosis, autophagy and microenvironment modulation in tumors (Ren et al., 2023). Recent studies (Chen T. et al., 2024; Sivalingam et al., 2024; Rui et al., 2025; Norbakhsh et al., 2022) focused on antitumor actinomycete metabolites emphasize that many compounds identified from plant-associated or otherwise unusual niches show differentiated mechanisms, such as multi-pathway cell-death induction and activity against resistant cell lines, making these organisms prime candidates for targeted mining of MDR-modulating chemotypes. Strategically, combining ecological screening of medicinal or stress-adapted plants with genome mining of endophytic actinomycetes and advanced metabolomics provides a rational route to uncover rare scaffolds and bioactivities that complement, rather than duplicate, the extensively mined soil-*Streptomyces* repertoire.

### 2.2 Actinomycetes metabolites: proven history in anticancer drug development

Actinomycetes have a long, well-documented history as one of the most productive sources of clinically important anticancer drugs, providing several first-in-class agents and inspiring many synthetic analogs (Selim et al., 2021; Rui et al., 2025). Their success in oncology underpins the rationale for continued mining of actinomycete metabolites, including those from endophytic and marine niches, as next-generation antitumor and anti-MDR leads.

Actinomycin D (dactinomycin), produced by *Streptomyces parvulus*, was the first microbial metabolite approved for cancer therapy and remains part of standard regimens for pediatric solid tumors such as Wilms’ tumor, rhabdomyosarcoma and Ewing sarcoma (Kapoor et al., 2022). It intercalates into DNA at guanine–cytosine–rich regions, inhibits RNA polymerase–mediated transcription and triggers p53-dependent apoptosis, illustrating how actinomycete antibiotics can be repurposed from antibacterial to antineoplastic use (Kapoor et al., 2022; Manikkam et al., 2024).

Mitomycin C, isolated from *Streptomyces caespitosus*, is a bioreductive alkylating agent used for gastrointestinal, breast and bladder cancers as well as in ophthalmology to prevent fibroproliferation. After reductive activation, it forms highly reactive mitosene intermediates that induce inter- and intra-strand DNA crosslinks, blocking replication and transcription and making it particularly effective in hypoxic tumor regions (Sethi et al., 2023).

Doxorubicin, daunorubicin, epirubicin and idarubicin are anthracycline antibiotics originally derived from Streptomyces species and are now among the most widely used chemotherapeutic agents worldwide. These molecules intercalate into DNA, poison topoisomerase II and generate reactive oxygen species, leading to double-strand breaks and apoptosis across a broad spectrum of malignancies. Clinical success with these drugs established actinomycetes as a premier source of potent DNA-targeting chemotypes, though dose-limiting cardiotoxicity has motivated continuous efforts to develop less toxic analogs and delivery systems (Hulst et al., 2022; van der Zanden et al., 2021).

Newer actinomycete-derived anthraquinones and benzo[a]naphthacenequinones, such as tsukubaricin and pradimicin U, exhibit strong *in vitro* cytotoxicity against lung, colon and breast cancer cell lines, sometimes with improved selectivity indices compared with classical anthracyclines, illustrating ongoing structural diversification from the same biosynthetic families (Bahrami et al., 2022; Menezes et al., 2023).

Rui et al. (2025) stated that clinically used and pre-clinical actinomycete anticancer metabolites span polyketides, non-ribosomal peptides, alkaloids, terpenoids and hybrid scaffolds. Beyond the historic DNA-interacting drugs, these include microtubule-targeting agents, protein synthesis inhibitors, enzyme inhibitors and compounds that modulate signaling pathways such as PI3K/Akt, MAPK and NF-κB. Marine actinomycetes in particular have yielded structurally novel metabolites capable of overcoming apoptosis resistance or restoring TRAIL sensitivity in triple-negative breast cancer models, highlighting their value for tackling drug-resistant disease.

Recent colorectal-cancer-focused analyses of actinomycete metabolites describe multiple agents with nanomolar to low-micromolar IC50 values and, in some cases, selectivity for tumor over normal cells, emphasizing that the pipeline of cytotoxic and targeted candidates from actinomycetes remains far from exhausted (Bahrami et al., 2022; Julianti et al., 2022).

A more oncology-focused overview on microbial metabolites similarly underscores that actinomycete products account for a disproportionate share of approved small-molecule anticancer drugs compared with other microbial groups, reinforcing their proven track record and the strong probability of identifying further therapeutically useful compounds from this lineage. Together, these data show that actinomycetes are not merely hypothetical sources of anticancer leads but have already furnished multiple cornerstone chemotherapeutics, spanning early DNA-directed antibiotics to modern topoisomerase inhibitors and structurally diverse next-generation candidates. This historical success provides a compelling foundation for prioritizing actinomycete, and especially endophytic and marine actinomycete, metabolites in contemporary campaigns aimed at discovering novel agents for drug-resistant and multidrug-resistant cancers. Some other actinomycetes derived compounds against MDR in cancer are depicted in Table 1.

**Table 1:** Actinomycetes-derived compounds against MDR in cancer.

Compound	Producing taxon	Host/Origin	Assay/Cell line	Potency (IC_50_/GI_50_ as reported)	Mechanism	References
2-Hydroxyethyl-3-methyl-1,4-naphthoquinone	*Actinoalloteichus cyanogriseus*	Not specified	MDA-MB-435 (breast cancer)	IC_50_ = 10.5 μM	Cytotoxicity (reported activity vs. invasive breast cancer cells)	Hwang et al. (2022)
ULDF-4	*Streptomyces bingchengensis* ULS-14	Marine-derived	MCF-7 (breast cancer)	IC_50_ = 0.0139 mg/mL (reported)	Cytotoxic; anti-proliferative in MCF-7	Xue et al. (2023)
ULDF-5	*Streptomyces bingchengensis* ULS-14	Marine-derived	MCF-7 (breast cancer)	IC_50_ = 2.176 mg/mL (reported)	Cytotoxic	Xue et al. (2023)
Coloraducin A5–A8 (A5, A6, A7, A8)	Streptococcus sp.	Not specified	MCF-7 (breast cancer)	IC_50_ = 3.7 nM, 14.7 nM, 133.0 nM, 10.5 nM (respective congeners)	Very potent cytotoxicity	Xue et al. (2023)
Sesbanimide A; Sesbanimides C–F	*Stappia indica*	Marine bacteria	MDA-MB-231 (breast cancer)	GI_50_ range 1.6 × 10^−7^ to 8.6 × 10^−9^ (very potent)	Polyketide cytotoxins—reported strong growth inhibition	Florêncio et al. (2022)
Tsukubaricin	*Streptomyces griseus*	Not specified	Multiple breast cancer cell lines (various human lines)	IC_50_: 24.0 nM	Anthracycline; activated from cryptic BGC (bioactivity-guided screening)	Wu et al. (2021)
Oligomycin derivatives	Streptomyces sp. FXY-T5	Marine-derived	DLD-1 (colorectal cancer)	Compounds with IC_₅₀_ < 10 μM	Antiproliferative activity; oligomycin class (mitochondrial ATP synthase inhibitors class are known)	Feng et al. (2024)
Rabelomycin	SCSIO LCY30	Not specified	SW480 (colon cancer) vs. NCM460 (normal intestinal cancer)	IC_50_ = 1.57 μM (SW480); normal cell IC_50_ = 7.05 μM → SI = 4.49	Selective cytotoxicity (higher toxicity to tumor vs. normal cells)	Feng et al. (2024)
Pradimicin U	FMUSA5-5^T^	Not specified	NCI-H187 (small cell lung cancer)	IC_50_ = 5.69 μg/mL	Dihydrobenzo[a]naphthacenequinone; selective antitumor in the reported assay	Duangupama et al. (2024)
Lanthomicin A	*Streptomyces chattanoogensis* L10	Not specified	A-549 (lung cancer)	IC_50_ = 0.17 μM	Pentangular polyphenolic polyketide; potent antiproliferative activity	Liu et al. (2022)
Crude methanol extract	Streptomyces sennicomposti GMY01	Marine/Soil	HeLa (cervical cancer), HTB (lung cancer)	IC_50_ = 27.31 μg/mL (HeLa); 33.75 μg/mL (HTB)	Crude extract with identified major component; molecular docking suggests autophagy/mTOR interactions for mannotriose	Widada et al. (2023)
Pigment fraction	Streptomyces tunisiensis W4MT573222	Not specified	HepG-2 (liver cancer), A549 (lung), PAN1	IC_50_ = 0.2 mg/mL (HepG-2); A549 = 1,114 μg/mL; PAN1 = 1,043 μg/mL	Pigment fraction shows selective cytotoxicity (higher potency vs. HepG-2)	Ibrahim et al. (2023)
Cervinomycin C analogs	Streptomyces sp. CPCC 204980,	Soil	HCT-116 colorectal cancer	Sub-nanomolar IC_50_ values; outperform many standards	High-potency cytotoxicity	Hu et al. (2020)
Lu01-M	Streptomyces sp. SCSIO LCY30	Marine-derived	PC-3, DU145, LNCaP (prostate cancer)	IC_50_ values of 1.03 ± 0.31, 2.12 ± 0.38, 1.27 ± 0.25 μg/mL respectively	Multimodal cytotoxic activity with induction of apoptosis and inhibition of cancer cell proliferation; detailed molecular mechanisms remain incompletely characterized	Rui et al. (2025)

## 3 Targeted bioprospecting: techniques for isolating MDR-active strains

Targeted bioprospecting aims to enrich for actinomycete strains with a high probability of producing bioactive metabolites, rather than randomly screening large isolate collections (Świecimska et al., 2023). This is especially important for discovering MDR-active compounds, where the goal is to capture phylogenetically diverse, chemically rich strains while minimizing redundancy. Modern strategies combine habitat selection, selective isolation, dereplication, high-content bioassays and genome or metabolome-guided prioritization to build focused libraries of “elite” strains (Ossai et al., 2022).

### 3.1 Habitat- and niche-directed sampling

A first level of targeting is the deliberate choice of extreme or specialized habitats that are likely to harbor unusual biosynthetic gene clusters, such as hyper-arid deserts, saline soils, deep-sea sediments, mangroves or medicinal-plant endospheres (Ahmad et al., 2024). Studies that sampled high-altitude Atacama desert soils, saline Indian soils and coniferous forest sites used standardized protocols to generate suspensions of environmental material, followed by plating on selective media and subsequent taxonomic analysis; this approach yielded large panels of rare or potentially novel actinomycete species with distinct bioactivity profiles (Shen et al., 2021). Similar bioprospecting campaigns in plant endophytes and marine sediments show that these “non-classical” niches significantly increase the proportion of strains that produce potent antibacterial or cytotoxic metabolites, including new scaffolds not observed in well-studied soil *Streptomyces* (Cabugao et al., 2022; Smith et al., 2018). For MDR-focused discovery, this niche targeting is attractive because stress-adapted communities often encode defense or signaling molecules that can translate into efflux modulation, DNA-damage responses or microenvironmental interactions in tumor systems (Labrie et al., 2022).

### 3.2 Selective isolation and enrichment

After sampling, selective isolation is used to suppress fast-growing bacteria and fungi and favor filamentous actinomycetes. Common approaches include heat pretreatment or dry-stamping of environmental material to kill non-spore-forming competitors, combined with the use of media containing antifungals, low nutrient levels or unusual carbon sources such as chitin, humic acids or complex plant polymers (Setlow and Christie, 2021). For marine and saline habitats, seawater-based media and elevated salt concentrations mimic *in situ* conditions and further enrich for adapted actinomycetes (Trenozhnikova et al., 2024). Differential pigmentation and colony morphology are often used as a first-pass classification tool (“color-group” strategy), enabling the selection of representative isolates from each morphotype while avoiding oversampling of common taxa (Rodrigues et al., 2023). These procedures generate culture collections that are more diverse taxonomically and metabolically than those obtained by standard nutrient-rich media alone, increasing the chance of isolating strains with novel MDR-relevant chemotypes.

### 3.3 Dereplication and taxonomic/phylogenetic targeting

To avoid re-discovering known producers, modern bioprospecting strategies incorporate dereplication at both the strain and metabolite level. At the strain level, clustering by colony color and morphology is now complemented by rapid methods such as 16S rRNA gene sequencing, molecular fingerprinting, and intact-cell MALDI-TOF mass spectrometry to group isolates into taxonomically meaningful units (Brauge et al., 2021). Representative strains from each cluster are then prioritized for detailed screening, greatly reducing redundancy in large libraries. At the metabolite level, HPLC or LC–MS fingerprinting is used to assess chemical output from small-scale fermentations; strains that produce overlapping metabolite profiles can be deprioritized, while those with distinct chromatographic or mass patterns are advanced (Zahn, 2017). This taxonomic and chemical dereplication strategy enriches for rare or novel actinomycete lineages whose metabolites are more likely to include new MDR-active scaffolds.

### 3.4 Bioassay-guided screening for MDR activity

Targeted bioprospecting for MDR-active metabolites depends on robust screening assays. Traditional agar diffusion and microbroth dilution tests against pathogenic bacteria are now supplemented by more sophisticated phenotypic screens (Salam et al., 2023). Examples include: co-culture assays against panels of clinically relevant “ESKAPE” pathogens (Tse et al., 2024), cell-based assays measuring DNA-damage responses (Collins et al., 2023), and reporter systems for efflux pump activity or membrane depolarization. Cultivation-based approaches usually fail to induce dormant biosynthetic gene clusters (BGCs), and cultivation-based approaches might favor those strains which are easy to cultivate. Culture-independent approaches have not been mentioned explicitly in the text but approaches like genome mining, metabolomics, metagenomics along with bioinformatics can be suggested here. Techniques such as co-culture, epigenetics, and regulatory network reengineering are among the techniques that help in induction of dormant BGCs (Mwangi et al., 2025). High-throughput micro-bioreactor systems allow parallel cultivation of hundreds of isolates under varying media conditions, with online or semi-automated sampling for bioassays (Potenza et al., 2025). For anticancer MDR, analogous strategies can be implemented using resistant tumor cell lines or efflux-reporter models to directly identify extracts that sensitize cells to standard drugs, inhibit transporter function or induce non-apoptotic cell death.

### 3.5 ONE STRAIN–MANY COMPOUNDS and cultivation-based activation of cryptic metabolites

A key lesson from recent work is that each actinomycete strain can produce widely different metabolite sets under different cultivation conditions. The “One Strain–Many Compounds” (ONE STRAIN–MANY COMPOUNDS) concept systematically varies media composition, pH, salinity, aeration and co-culture partners to activate silent biosynthetic pathways and expand the chemical space sampled from a given isolate. The One Strain–Many Compounds (OSMAC) method systematically alters culture conditions to activate silent pathways. High-throughput multi-well bioreactors with various media, pH, salinity, and coculturing are employed in particular for marine and saline endophytic strains. Genome-based techniques using antiSMASH and BAGEL4 software are used to annotate BGCs, making it easier to target them for activation. Metabologenomics matches metabolites to BGCs, helping with prioritization. Genomic and transcriptomic comparisons of regulatory genes affecting BGC expression allow for either genetic or chemical activation. High-throughput multi-well bioreactors with various media, pH, salinity, and coculturing are employed in particular for marine and saline endophytic strains. Genome-based techniques using antiSMASH and BAGEL4 software are used to annotate BGCs, making it easier to target them for activation. Metabologenomics matches metabolites to BGCs, helping with prioritization. Genomic and transcriptomic comparisons of regulatory genes affecting BGC expression allow for either genetic or chemical activation (Le Loarer et al., 2024). High-throughput implementation using multi-well bioreactors and automated LC–MS readouts has been particularly successful in marine and saline actinomycetes, where ONE STRAIN–MANY COMPOUNDS greatly increases the number of bioactive extracts and uncovers metabolites that would be missed under standard fermentations (Abdel-Razik et al., 2025). For MDR-oriented bioprospecting, ONE STRAIN–MANY COMPOUNDS-derived extracts can be screened specifically for efflux inhibition, synergy with known chemotherapeutics or activity against resistant tumor models. The concept of “synergy” seems to be used loosely since most interactions described can be additive rather than synergistic. A discussion of the difference between synergy, which means combined effects greater than the sum of individual effects, and additive effects, which means simple sums. Some mention of common approaches to measuring synergy.

#### 3.5.1 Genome- and metabolome-guided prioritization

Genome- and metabolome-guided prioritization in targeted bioprospecting leverages advanced omics and bioinformatics to systematically identify actinomycete strains most likely to produce novel MDR-active metabolites. After initial taxonomic screening and strain selection, researchers sequence whole genomes using long-read or multiplexed platforms, enabling the assembly of high-quality genomes and precise identification of biosynthetic gene clusters (BGCs), many of which remain silent under standard laboratory culture conditions (Landman et al., 2024). State-of-the-art algorithms such as antiSMASH and BAGEL4 are then used to annotate BGCs, forecast metabolite classes, and assess similarity to known clusters, allowing prioritization of strains with unusual or “orphan” clusters likely to yield new chemical entities (Suárez-Moo and Prieto-Davó, 2024). The endophytic actinomycetes live symptomless within different plant organs like roots, stems, leaves, and floral organs. The nature and functional capacity of such actinomycetes greatly depend on the type of host plant species, particular organ, and environmental conditions. Various ecological studies reveal that different types of plants and their organs contain different types of actinomycetes that have unique gene clusters involved in the biosynthesis of molecules. This approach helps to identify novel actinomycetes and novel metabolites. Simultaneously, metabolomic profiling (LC–MS/MS, molecular networking through platforms like GNPS) detects and organizes ions or compound families in extracts and linking observed masses (David et al., 2025). Integrated “metabologenomics” can connect metabolite features to biosynthetic gene signatures, especially when known peptide backbone structures or PKS domains are detected (Caesar et al., 2021). This cross-referencing accelerates dereplication, avoiding rediscovery of common compounds, and flags truly novel producers or metabolites for scale-up and deep characterization.

Some pipelines also apply comparative genomics and transcriptomics to identify regulatory genes responsible for BGC activation or silencing, uncovering sources of metabolic crypticity and further expanding the coverage of chemical diversity (Augustijn et al., 2024). Assigning function to silent BGCs (for example, those linked to MDR activity, efflux, or tumor microenvironment modulation) may require heterologous expression, chemical elicitors, or targeted mutagenesis to activate biosynthesis, coupling with subsequent bioactivity screening against MDR cancer cell lines or efflux/reporter systems to validate pharmacological relevance (Augustijn et al., 2024; Covington et al., 2021).

This integrated genome- and metabolome-guided approach allows researchers to prioritize not just strains, but also specific BGCs and metabolites, focusing resources on taxa and chemical families most likely to provide new MDR-active agents. Bioprospecting campaigns using these technologies have already revealed previously undetected clusters from actinomycetes inhabiting extreme or underexplored environments, and have enabled the discovery of new molecules with potent activity against resistant pathogens and cancer cell lines.

## 4 Anti-MDR arsenal from actinomycetes

### 4.1 Direct cytotoxicity against MDR cell lines

Recent systematic mapping of actinomycete metabolites shows multiple scaffolds with potent cytotoxicity and selectivity that make them strong candidates for overcoming MDR phenotypes (Chen T. et al., 2024; Bahrami et al., 2022). Anthracyclines such as tsukubaricin from *Streptomyces griseus* display sub-nanomolar to low-nanomolar inhibitory concentrations in breast and colon cancer models and often retain activity in cells with reduced doxorubicin sensitivity, suggesting partial evasion of classical anthracycline resistance mechanisms (Hulst et al., 2022).

For colorectal cancer models, Rui et al. (2025) collated a series of actinomycete metabolites, including rabelomycin, curacozole, cervinomycin analogs, oligomycins and tsukubaricin, with half-maximal inhibitory concentrations typically in the low-nanomolar to low-micromolar range. It is reported that there are some metabolites, like tsukubaricin and anthracycline analogs, which are found to have higher anti-proliferative efficacy compared to doxorubicin even in cells pre-treated with anthracyclines. Actinomycetes metabolites exhibit activity against MDR strains that are less sensitive to doxorubicin and other similar drugs implying that resistance has been partially circumvented. Nevertheless, a comparative study between parental and MDR strains is not widely conducted. Rabelomycin isolated from marine *Streptomyces SCSIO* LCY30, for example, showed an IC50 of about 1.6 μM against SW480 colon cancer cells, with a selectivity index around 4.5 relative to normal intestinal epithelial cells, indicating preferential tumor toxicity and suggesting that efflux or DNA repair mechanisms in normal cells do not negate its activity (Li et al., 2024). Cervinomycin C series analogs from *Streptomyces* sp. CPCC 204980 inhibited HCT-116 colon cancer cells with sub-nanomolar IC50 values, demonstrating much higher potency than many standard chemotherapeutics and highlighting the capacity of actinomycete polyketides to bypass or outcompete existing resistance pathways (Hu et al., 2020).

Many of these metabolites induce multifaceted cell death programs that are relevant for MDR settings. Lu01-M, an *Streptomyces*-derived compound triggers apoptosis, necrosis, autophagy, endoplasmic reticulum stress, G2/M arrest and DNA damage while impairing colony formation and migration, which is advantageous because MDR cells often survive by selectively blocking single death pathways (Lin et al., 2021). Pigment mixtures from *Streptomyces tunisiensis* W4MT573222 were cytotoxic to hepatocellular, lung and pancreatic cancer lines with modest IC50 values while exhibiting about two-fold higher IC50 in normal Vero cells, again reflecting some therapeutic window that could be exploited in resistant disease (Ibrahim et al., 2023).

Collectively, these data indicate that secondary metabolites from endophytic and marine actinomycetes comprise a rich portfolio of highly potent cytotoxins that retain activity in models with reduced sensitivity to classical agents, display favorable selectivity indices and frequently activate multiple, parallel cell death programs, key features for tackling multidrug-resistant tumors. However, some parts indicate that decreases in migration or colony formation could indicate impact on EMT, cancer stemness or TME remodeling. The data underlying these observations are predominantly based on the inhibition of phenotype, without validation of mechanisms for the complicated biological processes. Moderation is needed in describing these as evidence of effects rather than as conclusive findings.

### 4.2 Resistance-reversal modulators (efflux and survival pathways)

Beyond direct cytotoxicity, several actinomycete-associated metabolites function as functional MDR modulators by altering drug transport and intracellular signaling (Wink et al., 2012). Clinically used actinomycete-derived drugs such as mitomycin C and anthracyclines enhance the activity of co-administered chemotherapeutics by interfering with efflux transporters or downstream survival cascades (Kapoor et al., 2022; Giurini et al., 2024). For example, tsukubaricin and related anthracycline analogs exhibit stronger antiproliferative effects than doxorubicin in breast cancer cells, including those with prior exposure to anthracyclines, implying alterations in cellular uptake, DNA binding kinetics or efflux susceptibility (Wu et al., 2021).

Complementary MDR reviews on natural products describe multiple microbial-derived agents that directly or indirectly inhibit ATP-binding cassette transporters such as P-glycoprotein (ABCB1), BCRP (ABCG2) and MRPs (Martins-Gomes and Silva, 2023). These compounds typically increase intracellular accumulation of partner drugs, restore pro-apoptotic signaling and down-regulate MDR gene expression, thereby sensitizing resistant cells to standard chemotherapy. Recent work on human gut Actinobacteria further demonstrates that secreted metabolites can inhibit P-gp efflux and significantly enhance the cytotoxicity of doxorubicin *in vitro*, providing mechanistic proof that actinobacterial products can modulate transporter-mediated MDR in a clinically relevant way (Kyaw et al., 2024). Together, these data position actinomycete metabolites as promising building blocks for next-generation resistance-reversal combinations that simultaneously attack tumor survival networks and transport barriers.

### 4.3 Targeting microenvironment and cancer stemness

The tumor microenvironment and cancer stem cell (CSC) populations critically sustain MDR by shielding tumor cells from drugs, promoting epithelial–mesenchymal transition (EMT), and up-regulating efflux transporters and DNA repair (Mengistu et al., 2024). Reviews on actinomycete metabolites (Chen T. et al., 2024; Chinnathambi et al., 2023; Bahrami et al., 2022) highlight several compounds that affect processes linked to invasion, metastasis and TME crosstalk, such as inhibition of migration, colony formation and angiogenesis, although detailed *in vivo* microenvironmental profiling remains limited. For instance, the multi-target compound Lu01-M not only induces diverse cell death mechanisms but also suppresses cell migration, suggesting interference with EMT-related pathways that are closely tied to CSC plasticity and drug tolerance (Rui et al., 2025).

Broader TME-focused natural-product’s literature shows that microbial metabolites can modulate extracellular ATP levels, inflammatory cytokine signaling and stromal interactions, which in turn regulate EMT, CSC formation and ABC transporter activity (Dias et al., 2021). Actinomycete-derived antibiotics such as mitomycin C and certain anthracyclines have documented anti-angiogenic and immunomodulatory effects, reducing vascular support and improving immune recognition mechanisms that may indirectly reduce MDR by depriving resistant clones of protective niches. The paper often mistakenly associates the discovery of novel biosynthetic gene clusters through genome mining with evidence of MDR-related bioactivity. Genome novelty is certainly an interesting beginning, but does not necessarily mean that there is pharmacological activity. Making the differentiation between the two could be beneficial for the precision of results (Pandey and Gupta, 2024; Hulst et al., 2022). Integrating these insights, actinomycete metabolites can be framed not only as cytotoxic or transporter-modulating agents, but also as potential tools to “reprogram” the microenvironment and CSC compartment, thereby attacking MDR at its ecological roots.

## 5 Mechanisms of action against resistance pathways

### 5.1 Efflux pump inhibition

ATP-binding cassette (ABC) transporters such as P-glycoprotein (P-gp/ABCB1), breast cancer resistance protein (BCRP/ABCG2) and multidrug resistance-associated proteins (MRPs) are central determinants of multidrug resistance because they actively expel structurally diverse cytotoxic drugs and targeted agents, reducing intracellular exposure below lethal thresholds (Chen R. et al., 2024; Zattoni et al., 2022). Recent reviews emphasize that natural products can counter this mechanism by directly inhibiting transporter function, suppressing expression of ABC genes or altering upstream signaling pathways that regulate transporter transcription (Figure 1) (Ibrahim et al., 2023; Augustijn et al., 2024; Shen et al., 2011).

**Figure 1:**
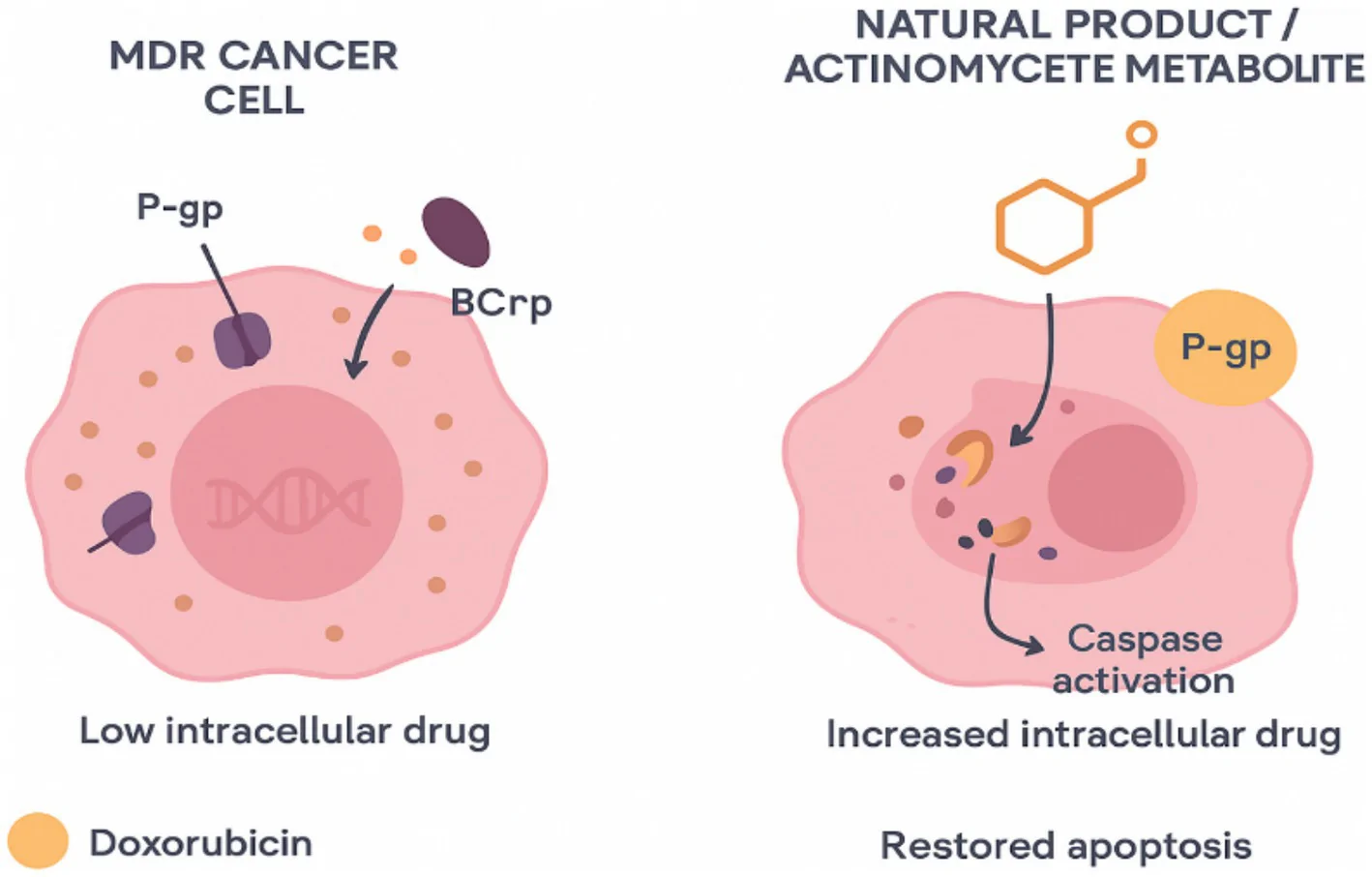
Inhibition of efflux pumps by natural products and actinomycete metabolites restores chemotherapeutic sensitivity in MDR cancer cells.

Zou et al. (2024) highlight multiple phytochemicals, such as flavonoids, terpenoids and alkaloids, that restore sensitivity of P-gp-overexpressing cells by reducing MDR1 mRNA and protein expression, inhibiting ATPase activity and increasing intracellular accumulation of drugs like doxorubicin, paclitaxel or 5-fluorouracil. Chen T. et al. (2024) similarly report compounds such as curcumin, resveratrol and baicalein that down-regulate ABCB1 or ABCG2 via modulation of NF-κB, PI3K/Akt and MAPK signaling, thereby reversing resistance in diverse tumor models. Overlaying these paradigms with actinomycete data, Rui et al. (2025) in their systematic review notes that several actinomycete-derived agents, including clinically established anthracyclines and mitomycin C, remain active in cells with increased efflux capacity, and those newer metabolites, together with gut Actinobacteria-secreted products, can enhance intracellular drug levels by interfering with P-gp-mediated transport. *In vitro*, metabolites from human gut Actinobacteria have been shown to inhibit P-gp function and markedly potentiate doxorubicin cytotoxicity, providing proof-of-concept that actinobacterial metabolites can serve as transporter-targeted MDR modulators (Kyaw et al., 2024).

Multidrug-resistant (MDR) cancer cells overexpress efflux pumps such as P-glycoprotein (P-gp/ABCB1) and BREAST CANCER RESISTANCE PROTEIN (ABCG2), which export chemotherapeutic agents (e.g., doxorubicin) and reduce intracellular drug accumulation, leading to treatment failure. A number of actinomycete-derived metabolites have been identified to possess cytotoxic activity toward MDR cancer cells, but there is little evidence that these metabolites can modulate transporters such as the P-glycoprotein (P-gp/ABCB1) or breast cancer resistance protein (BREAST CANCER RESISTANCE PROTEIN/ABCG2). Most of the actinomycetes are known for their cytotoxic or antiproliferative activities, whereas a small number have been found to modulate the MDR mechanisms, like increasing intracellular r drug uptake or inhibiting transporter proteins. For instance, microbial metabolites derived from Actinobacteria of the human gut microbiome have been found to inhibit the efflux of P-gp and increase the cytotoxicity of doxorubicin. Natural compounds, particularly those derived from actinomycetes, can inhibit efflux pump function, allowing intracellular drug retention, activation of caspase-dependent apoptosis, and resensitization to chemotherapeutics.

### 5.2 Inducing non-apoptotic cell death

Zou et al. (2024) stresses that MDR tumors often evade classical apoptosis, making alternative modes of regulated cell death, such as ferroptosis, necroptosis and autophagy-dependent death, attractive therapeutic targets. Multiple natural products, including certain polyphenols and terpenoids, trigger ferroptosis by depleting glutathione, inhibiting GPX4 or promoting lipid peroxidation (Zhang and Xie, 2024), while others promote necroptosis through RIPK1/RIPK3/MLKL activation (Liang et al., 2023) or induce lethal autophagy by modulating Beclin-1 and mTOR signaling (Cao et al., 2024), thereby killing cells that are resistant to caspase-dependent apoptosis (Figure 2).

**Figure 2:**
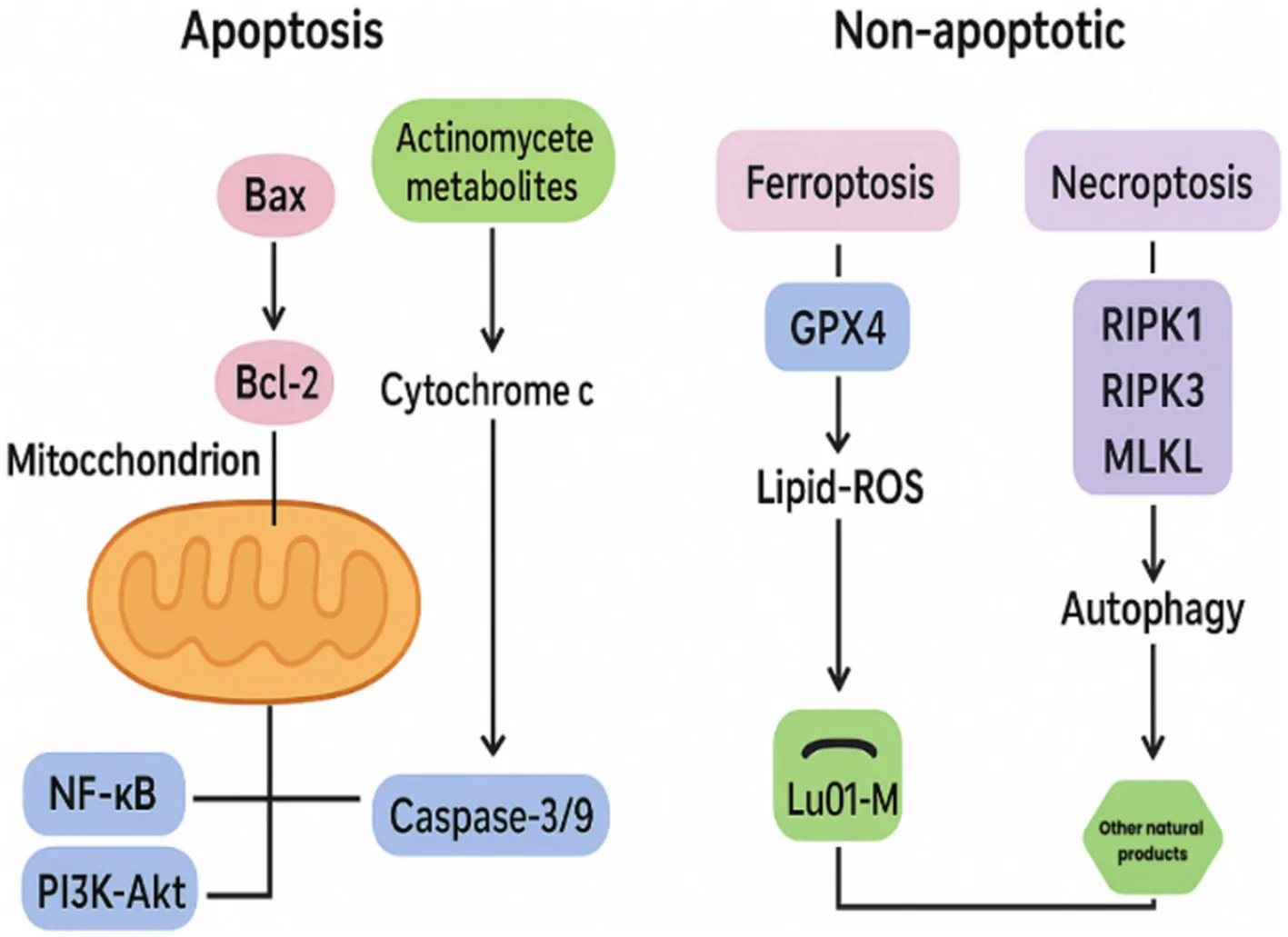
Modulation of apoptotic and non-apoptotic cell death pathways by natural products and actinomycete metabolites.

Within this framework, Rui et al. (2025) in his compilation of actinomycete metabolites reveals several candidates capable of engaging non-apoptotic death pathways. Lu01-M represents a prototypical example, inducing a complex cytotoxic phenotype that includes apoptosis, necrosis and autophagy alongside endoplasmic reticulum stress, G2/M arrest and DNA damage, features consistent with activation of multiple regulated death programs in parallel (Rui et al., 2025; Bahrami et al., 2022). Such poly-mechanistic behavior is particularly desirable in MDR settings, where redundancy of survival pathways frequently undermines single-mode interventions. Although detailed ferroptosis or necroptosis signatures have not yet been fully characterized for most actinomycete metabolites, the emerging evidence that these compounds can simultaneously trigger apoptosis-independent death routes points to a promising avenue for future mechanistic and translational work.

Natural products, including actinomycete-derived compounds, restore cancer cell sensitivity by rebalancing cell death signals. These agents up-regulate pro-apoptotic factors (Bax, Bak, caspase-3/9) and suppress anti-apoptotic pathways (Bcl-2, NF-κB, PI3K/Akt), while also inducing non-apoptotic modalities, such as ferroptosis, necroptosis, and autophagy, to overcome apoptosis resistance and enable effective tumor cell killing.

### 5.3 Modulation of pro- and anti-apoptotic pathways

Chen T. et al. (2024) and Zou et al. (2024) review that many natural products counter MDR by re-balancing apoptotic signaling, typically by enhancing pro-apoptotic mediators and suppressing anti-apoptotic or survival pathways. These compounds commonly up-regulate Bax, Bak and cleaved caspase-3/9, down-regulate Bcl-2 and Bcl-xL (Figure 2), and inhibit pro-survival pathways such as PI3K/Akt, NF-κB and STAT3, thereby restoring susceptibility to DNA-damaging agents and targeted therapies even in resistant cell lines.

Rui et al. (2025) actinomycete survey indicates that many secondary metabolites from *Streptomyces* and related genera exert their antitumor effects through exactly these axes. Several polyketides and non-ribosomal peptides were reported to induce mitochondrial-dependent apoptosis with increased Bax/Bcl-2 ratios, cytochrome c release and caspase activation, while simultaneously inhibiting proliferation-linked pathways such as PI3K/Akt or MAPK. For instance, anthracycline-type metabolites derived from actinomycetes generate DNA double-strand breaks, leading to p53 activation and downstream apoptosis (Hulst et al., 2022), whereas other compounds trigger caspase-dependent cell death without significant genotoxicity, suggesting more direct modulation of Bcl-2 family proteins or death receptors (Jiao et al., 2013). These data support a model in which actinomycete metabolites not only kill MDR cells directly but also resensitize them to conventional drugs by rewiring the balance between pro- and anti-apoptotic signals.

### 5.4 Tumor microenvironment and stemness

Zhang and Xie (2024) and Zhang H. et al. (2024) emphasize that the tumor microenvironment (TME) including immune cells, stromal fibroblasts, vasculature and extracellular matrix play a pivotal role in fostering MDR by limiting drug penetration, secreting pro-survival cytokines and nurturing cancer stem cells (CSCs) (Figure 3). Their review documents how natural products can remodel the TME by re-programming tumor-associated macrophages, enhancing cytotoxic T-cell activity, normalizing abnormal vasculature and disrupting pro-fibrotic stromal signaling, ultimately improving drug delivery and diminishing microenvironment-mediated resistance. In parallel, natural compounds can reduce CSC frequency and plasticity by modulating EMT-related transcription factors, epigenetic regulators and pathways such as Wnt/β-catenin, Notch and Hedgehog.

**Figure 3:**
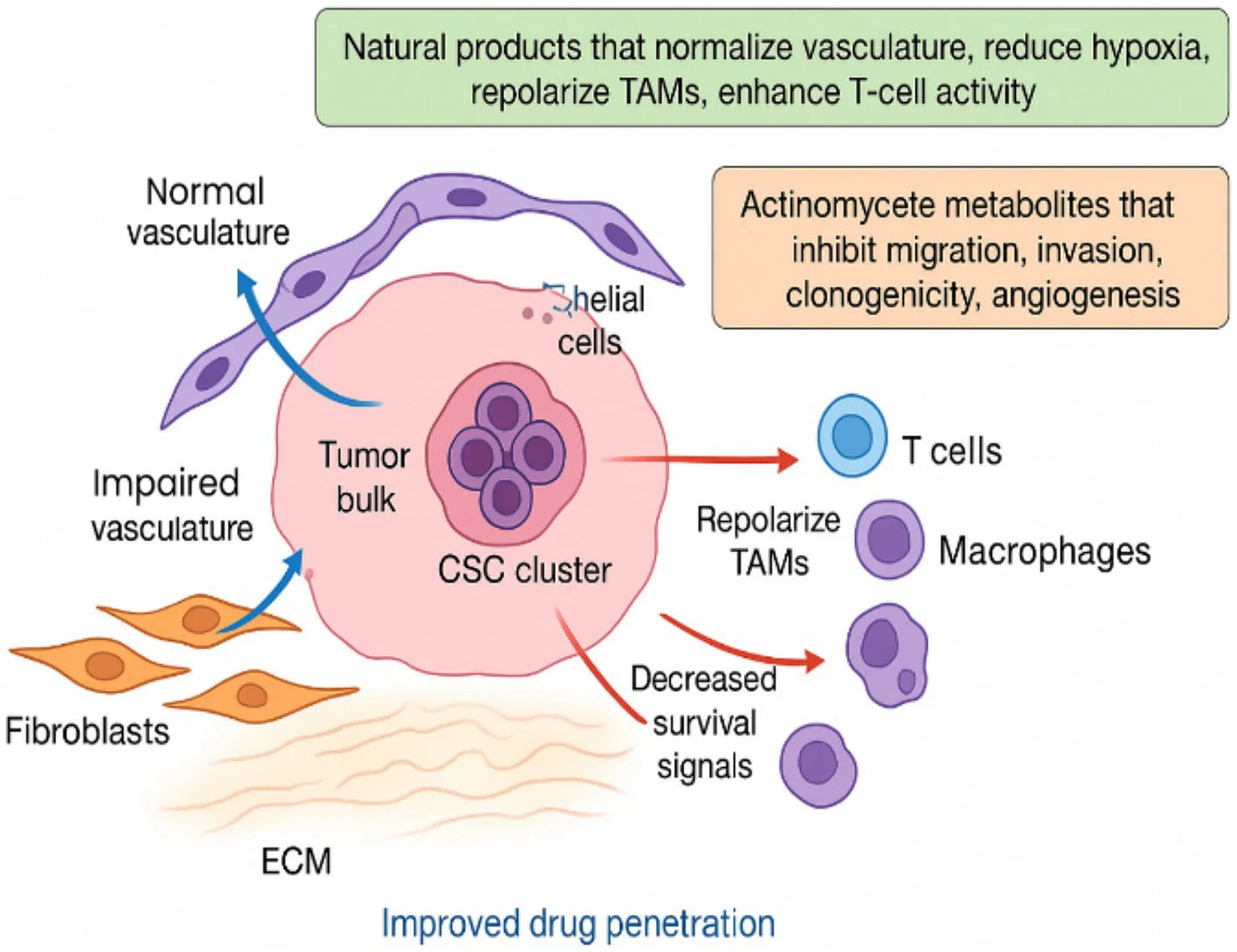
Tumor microenvironment and cancer stemness modulation by natural products and actinomycete metabolites.

Rui et al. (2025) identifies multiple metabolites with the capacity to interfere with microenvironmental and stemness-related processes, even if explicit CSC assays are still rare. Several compounds inhibit cancer cell migration, invasion and colony formation, phenotypes that are closely linked to EMT and metastatic CSC behavior. Lu01-M again stands out by suppressing migration and clonogenic growth in addition to its multi-modal cytotoxic effects, suggesting that it may attenuate EMT and limit the emergence of resistant CSC subpopulations (Bahrami et al., 2022). Moreover, some actinomycete-derived drugs, such as mitomycin C and certain anthracyclines, possess anti-angiogenic and immunomodulatory properties that reduce vascular support and can enhance immune-mediated tumor clearance, thereby indirectly lowering the threshold for successful chemotherapy in MDR settings (Pourmadadi et al., 2023). As mechanistic studies expand, these observations support the view that actinomycete metabolites can be integrated into broader natural-product strategies aimed at dismantling the ecological and stemness-based foundations of drug resistance.

Natural products and actinomycete metabolites disrupt the tumor microenvironment (TME) and cancer stem cell (CSC) niches, diminishing stromal protection, inhibiting angiogenesis, and reversing epithelial–mesenchymal transition (EMT). These interventions enhance anti-tumor immunity, reduce CSC-mediated resistance, and promote durable responses in multidrug-resistant cancers.

## 6 Translational potential and future directions

### 6.1 Translational challenges

Current MDR and TME-focused reviews converge on several bottlenecks that limit clinical use of natural-product MDR modulators despite abundant *in vitro* efficacy. First, many flavonoids, polyphenols and alkaloids suffer from poor aqueous solubility, chemical instability and low oral bioavailability, resulting in unfavorable pharmacokinetic and pharmacodynamic (PK/PD) profiles and making it difficult to reach concentrations required to inhibit efflux pumps or reprogram the tumor microenvironment in patients (Naeem et al., 2022). Second, pleiotropic target profiles can translate into off-target toxicity, particularly cardiotoxicity, hepatotoxicity or neurotoxicity, when agents interact with physiological ABC transporters, detoxification enzymes or immune pathways in healthy tissues (Tosca et al., 2021). Interactions of herbal drugs but this information is basically founded on phytochemical studies rather than on actinomycetes metabolites. It will be clear to state this aspect and distinguish the findings from microorganisms and plants separately. A third translational barrier is the potential for herb-drug interactions when natural-product MDR modulators are combined with standard chemotherapy or targeted agents, because many such compounds modulate cytochrome P450 enzymes, drug transporters and coagulation pathways, raising concerns about unpredictable exposure and safety (Pintado et al., 2023). Finally, both Chen T. et al. (2024) and Zou et al. (2024) emphasize that, compared to the extensive pre-clinical literature, formal phase I–III clinical trials of natural-product MDR modulators remain scarce, often limited to small cohorts or traditional formulations, and rarely incorporate robust biomarkers of MDR or TME remodeling. TME-oriented reviews add that heterogeneity of stromal and immune landscapes, as well as context-dependent effects of immunomodulatory natural products, complicate the extrapolation of pre-clinical findings into durable clinical benefit.

### 6.2 Strategies to overcome barriers

To address these challenges, several complementary strategies are emerging from the MDR and TME literature. Semi-synthetic optimization and medicinal chemistry have generated derivatives such as improved flavonoids and bisbenzylisoquinoline alkaloids (for example, optimized analogs of tetrandrine) with enhanced solubility, higher potency against P-gp or BREAST CANCER RESISTANCE PROTEIN, and reduced off-target cardiotoxicity, while preserving the ability to resensitize resistant tumor cells. Rational modification of pharmacophores guided by structure–activity relationships, computational modeling and absorption, distribution, metabolism, excretion, and toxicity (ADMET) profiling is increasingly used to fine-tune lipophilicity, metabolic stability and transporter affinity (Nguyen et al., 2021; Johnson et al., 2024).

Nanotechnology offers a second major avenue by packaging natural-product modulators into liposomes, polymeric nanoparticles, micelles or hybrid systems that improve aqueous dispersibility, prolong circulation time and concentrate drugs within tumors via enhanced permeability and retention or active targeting (Arunachalam et al., 2023). TME-focused reviews describe nanocarriers co-loading chemotherapeutics with natural products such as curcumin or quercetin, achieving synergistic killing of resistant cells while minimizing systemic toxicity and concurrently modulating angiogenesis, hypoxia and immune cell function (Parikh and Shah, 2024; Bauer, 2021). Combination regimens represent a third pillar: MDR reviews document numerous pre-clinical examples where flavonoids, terpenoids or coumarins restore sensitivity to cisplatin, doxorubicin or 5-fluorouracil by simultaneously inhibiting efflux, reinstating apoptosis and attenuating pro-survival signaling, with promising early-phase clinical trials for certain optimized derivatives. The article frequently makes references to well-understood MDR modification through plant-based flavonoids and alkaloids, and then applies the same principles to compounds produced by actinomycetes. There is no biochemical evidence available with regards to the mode of action of such actinomycete products in terms of transporter inhibition or modulation of MDR genes (Zou et al., 2024; Pintado et al., 2023). Finally, biomarker-driven patient selection—using signatures of ABC transporter expression, hypoxia markers, immune infiltration patterns or epigenetic alterations (Upadhyay et al., 2023) has been proposed to identify subgroups most likely to benefit from specific natural-product MDR modulators, although this approach is only beginning to be incorporated into trial design.

## 7 Perspective: endophytic actinomycetes as next-generation MDR modulators

Within this translational landscape, endophytic actinomycetes represent a relatively underexplored but strategically attractive subset of natural-product sources. Rui et al. (2025), Chen T. et al. (2024) and Bahrami et al. (2022) catalog a broad spectrum of actinomycete-derived metabolites (including polyketides, non-ribosomal peptides and hybrid scaffolds) that exhibit nanomolar cytotoxicity, multi-pathway cell-death induction, and in some cases transporter modulation or anti-angiogenic activity, yet only a small fraction of these compounds have been advanced beyond *in vitro* testing. Their chemical diversity, presence in plant-associated niches and amenability to genome mining and synthetic biology make them ideal candidates for rational MDR-oriented discovery programs.

A forward-looking pipeline could therefore prioritize endophytic actinomycetes using a multi-stage workflow: genome mining of biosynthetic gene clusters in endophytic isolates to predict novel scaffolds; targeted metabolite isolation and structural elucidation; systematic profiling of MDR-relevant mechanisms *in vitro* (including efflux inhibition, apoptosis/non-apoptotic death, TME and stemness modulation); followed by *in vivo* validation in xenograft and syngeneic models that recapitulate resistant disease. Compounds showing favorable efficacy, tolerability and PK/PD properties would then enter a medicinal-chemistry phase focused on semi-synthetic optimization, nanoparticle-based formulation and rational combination with standard chemotherapy or immunotherapy in biomarker-defined patient subsets. Implemented systematically, this pipeline positions endophytic actinomycete metabolites as a next-generation arm of natural-product-based MDR therapeutics, complementing plant-derived modulators and expanding the repertoire of tools available for precision management of drug-resistant cancer.

## 8 Limitations

Experimental models used in different studies are also not uniform, involving different cell lines, different assay formats, and different endpoints. IC50, GI50, and other parameters are used for potency without any unified conditions. In addition, there is less evidence on MDR mechanisms mediated by endophytic actinomycetes in comparison to their antibacterial or anticancer effects in general. Pharmacokinetics limitations, off-target effects, and lack of biomarker-based clinical trials are also a problem. The integrated genomic and metabolomics strategy discussed above is very potent but resource intensive. The cost involved, along with the need for next generation sequencing platforms and bioinformatics capabilities, could be bottlenecks.

## 9 Conclusion

Drug-resistant cancer remains a central cause of therapeutic failure worldwide, driven by a complex interplay of transporter-mediated efflux, deregulated cell-death pathways, protective tumor-microenvironmental niches and cancer stemness. Natural products have demonstrated substantial potential to counter these mechanisms, yet only a small subset has progressed to well-designed clinical trials because of pharmacokinetic limitations, safety concerns and the lack of biomarker-guided development. Evidence summarized in this review indicates that endophytic actinomycetes constitute a particularly rich but underexplored source of metabolites capable of exerting potent cytotoxic effects in MDR models, modulating ABC transporters, re-balancing apoptotic and non-apoptotic death programs, and reshaping the tumor microenvironment and stem-like compartments.

To convert this chemical diversity into tangible clinical benefit, future work should adopt an integrated pipeline that couple’s genome mining and metabolomics of endophytic actinomycetes with phenotypic and target-based MDR assays, followed by *in vivo* validation in resistance-focused models and iterative semi-synthetic or formulation optimization. Embedding these efforts within rational combination strategies and biomarker-driven patient selection will be essential to minimize toxicity and maximize the probability of clinical success for actinomycete-derived MDR modulators. Positioned within this framework, endophytic actinomycetes are poised to contribute a new generation of mechanistically informed agents that complement existing therapies and help close the gap between pre-clinical promise and durable control of drug-resistant malignancies.

## Data Availability

The original contributions presented in the study are included in the article/supplementary material, further inquiries can be directed to the corresponding author.
